# A Simple Logistic Regression Model for Predicting the Likelihood of Recurrence of Atrial Fibrillation Within 1 Year After Initial Radio-Frequency Catheter Ablation Therapy

**DOI:** 10.3389/fcvm.2021.819341

**Published:** 2022-01-27

**Authors:** Sixiang Jia, Haochen Mou, Yiteng Wu, Wenting Lin, Yajing Zeng, Yiwen Chen, Yayu Chen, Qi Zhang, Wei Wang, Chao Feng, Shudong Xia

**Affiliations:** ^1^Department of Heart Center, The Fourth Affiliated Hospital of Zhejiang University School of Medicine, Yiwu, China; ^2^Department of Orthopedic Surgery, The Second Affiliated Hospital of Zhejiang University School of Medicine, Hangzhou, China

**Keywords:** the recurrence of atrial fibrillation, radio-frequency catheter ablation, prognosis, Lasso regression, logistic regression prediction

## Abstract

**Background:**

The clinical factors associated with the recurrence of atrial fibrillation (Af) in patients undergoing catheter ablation (CA) are still ambiguous to date.

**Purpose:**

1. To recognize preoperative serologic factors and clinical features associated with Af recurrence after the first ablation treatment. 2. To Develop a Logical Regression Model for Predicting the Likelihood of Recurrence Within 1 Year After the Initial Radio-Frequency Catheter Ablation (RFCA) Therapy.

**Methods:**

Atrial fibrillation patients undergoing RFCA at our institution from January 2016 to June 2021 were included in the analysis (*n* = 246). A combined dataset of relevant parameters was collected from the participants (clinical characteristics, laboratory results, and time to recurrence) (*n* = 200). We performed the least absolute shrinkage and selection operator (Lasso) regression with 100 cycles, selecting variables present in all 100 cycles to identify factors associated with the first recurrence of atrial fibrillation. A logistic regression model for predicting whether Af would recur within a year was created using 70% of the data as a training set and the remaining data to validate the accuracy. The predictions were assessed using calibration plots, concordance index (C-index), and decision curve analysis.

**Results:**

The left atrial diameter, albumin, type of Af, whether other arrhythmias were combined, and the duration of Af attack time were associated with Af recurrence in this sample. Some clinically meaningful variables were selected and combined with recognized factors associated with recurrence to construct a logistic regression prediction model for 1-year Af recurrence. The receiver operating characteristic (ROC) curve for this model was 0.8695, and the established prediction model had a C-index of 0.83. The performance was superior to the extreme curve in the decision curve analysis.

**Conclusion:**

Our study demonstrates that several clinical features and serological markers can predict the recurrence of Af in patients undergoing RFCA. This simple model can play a crucial role in guiding physicians in preoperative evaluation and clinical decision-making.

## Introduction

Atrial fibrillation (Af) is currently one of the most common cardiac arrhythmias. Mortality in patients with Af is two times as high as in people with normal sinus heart rhythm (1). If left untreated, it can lead to complications, such as heart palpitations, chest tightness, heart failure, thrombosis, stroke, and cognitive impairment, which can seriously affect the quality of life of a patient. Catheter ablation (CA) is more effective than drug therapy in treating atrial fibrillation (2). However, 30–50% of patients undergoing CA suffer from a recurrence within 1 year of the procedure (3).

Many scholars have researched the high recurrence rate of Af after CA, but the underlying mechanism remains elusive. Some theories describe cardiomyocytes and myocardial sleeves in the pulmonary veins to produce ectopic autoregulation, triggering atrial fibrillation (4). It does not have a specific mechanism, such as atrial flutter, which is a folding ring dependent on the inferior vena cava-tricuspid isthmus (5). However, there is a low risk of atrial flutter recurrence after CA.

Multiple studies have been carried out on the high recurrence rate of Af. Based on the treatment experience of our team, it is now believed that the type of Af determines the treatment prognosis in cases where the mechanism of Af is unclear. Differences in the treatment of paroxysmal Af vs. persistent Af have been noted (6). The type of Af depends largely on the patient's own underlying disease status. The progression of Af causes remodeling of the atrial model, and the elevation of serological indicators secreted by atrial muscle cells allows for the assessment of cardiac function and prognosis after CA. However, there is currently no mathematical model quantifying the recurrence rate of Af.

Several studies have reported using differences in CA procedures to predict early recurrence of Af (7). Bisbal F et al. used the morphology of left atrial remodeling to predict outcomes after ablation. Regrettably, these intraoperative parameters of interest do not provide patients and clinicians with preoperative decisions (8).

In this general context, we are more interested in quantifying preoperative parameters concerning recurrence outcomes to provide clinical guidance before ablation treatment and minimize recurrence rates in subsequent patients. Few similar studies have been reported.

Since our center specializes in radio-frequency catheter ablation (RFCA), we aimed to raise awareness of clinical features and laboratory factors related to RFCA and construct logistic regression models to predict whether Af will recur within 1 year following RFCA.

## Methods

### Study Design and Patients

The Ethics Committee of the Fourth Affiliated Hospital of Zhejiang University School of Medicine review board approved this retrospective study, which was conducted in accordance with the principles of the Helsinki Declaration. All medical record data were collected at our medical centers. Inpatients with Af who underwent CA at our hospital were included.

**Figure 1:**
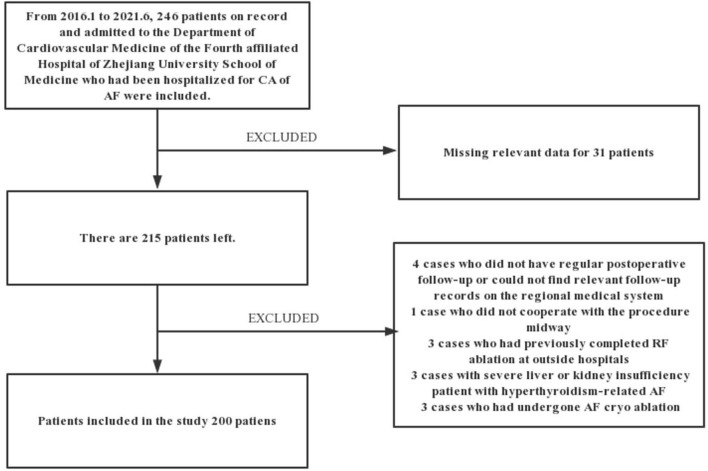
Flowchart of the study.

**Figure 2:**
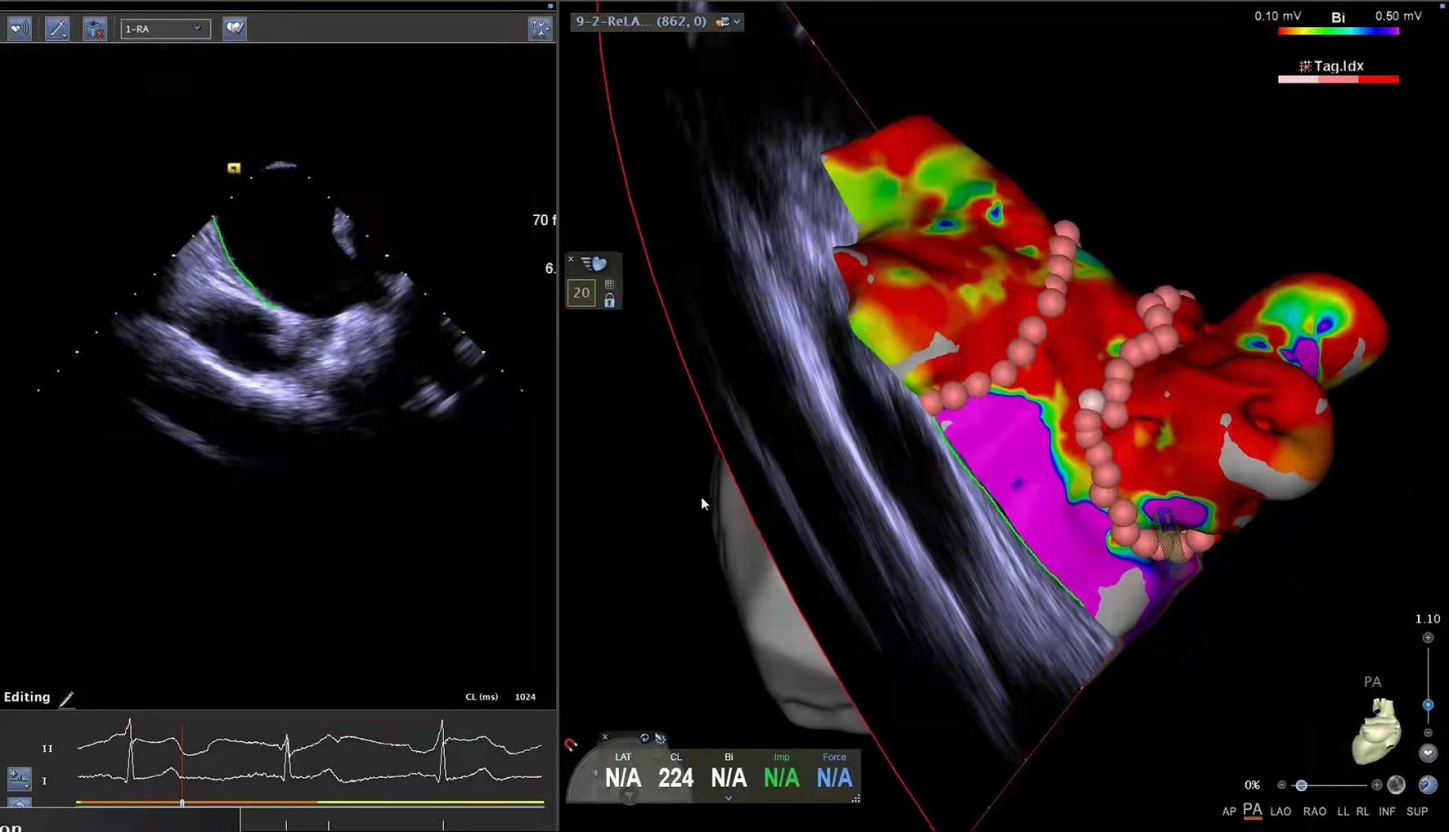
Radio-frequency ablation process details.

This is a retrospective study based on the records of patients. From January 2016 to June 2021, 246 patients admitted to the Department of Cardiovascular Medicine of the Fourth affiliated Hospital of Zhejiang University School of Medicine hospitalized for CA of Af were included in this study. Considering the nature of Af diagnosis and the need for data analysis, we excluded 4 cases due to lack of regular follow-up after the procedure or missing records in the medical system of the hospital. Patients that did not complete the procedure (*n* = 1), having previously completed CA at other hospitals (*n* = 3), with severe liver or renal insufficiency (*n* = 3), with hyperthyroidism-related Af (*n* =1), having undergone frozen Af ablation (*n* = 3), and patients with missing data on the inpatient system (*n* = 31) were excluded from this study. The remaining 200 patients met the inclusion criteria, were diagnosed with Af, and completed the procedure at our institution with at least 3 months of follow-up. Figure 1 shows the detailed flowchart of our study. We also provide relevant ablation details as shown in Figure 2. A comprehensive dataset of relevant parameters (including clinical factors, laboratory factors, ancillary tests, and follow-up information) was collected in patients who met the criteria. Two experienced electrophysiologists performed all the procedures.

The exclusion criteria for the study are as follows:

(1) Have received relevant CA treatment in other medical institutions.
(2) Reversible causes of Af: fever, severe infections, electrolyte disturbances, alcohol consumption (holiday heart syndrome), hyperthyroidism, etc. (9–11).
(3) Acute hepatic or renal insufficiency or inability to complete the procedure due to anticoagulation risk or acute chest pain or other systemic diseases.
(4) Lack of detailed information related to ablation.
(5) Absence of clinical features and preoperative laboratory tests.
(6) No regular follow-up or less than 3 months of follow-up.

### Variables, Outcome Measures, Information Source, and Bias

Our primary outcome was the recurrence of Af in patients who had undergone RFCA for the first time at our center during the follow-up period. The time between initial ablation and recurrence was measured (similar to progression-free survival and except for deaths). The study cases included follow-up until October 2021. Recurrence was defined as an episode of Af captured by a 12-lead ECG or 24-h ambulatory ECG completed at a regular healthcare facility.

The secondary outcome was the recurrence rate within 1 year after the procedure.

The clinical characteristics obtained from the electronic medical record system of the patient were evaluated and reconfirmed at follow-up, including the previous underlying disease of the patient, duration of Af symptoms and initial CA therapy time, procedure method (cryoablation or RFCA), the reappearance of preoperative symptoms, and the time until recurrence of Af. Cardiac ultrasound reports were evaluated to assess the left atrial diameter and left ventricular ejection fraction. Similarly, the type of Af (persistent or paroxysmal) and whether other arrhythmias combined were identified at follow-up, as the type of Af largely determines the post-ablation prognosis (6). Additionally, the CHA2DS2-VASc and HAS-BLED scores were assessed (12, 13). To mitigate the influence of the ablation procedure on this evaluation, we recorded the procedure time and the ablation power used as well.

Routine blood tests, blood biochemistry, cardiac enzyme profile, B-type natriuretic peptide (BNP), and troponin levels were obtained from our laboratory department. All patients in this study were tested for blood markers on admission to the hospital. The most recent preoperative test results were selected for patients with multiple preoperative tests. Cardiac ultrasound reports were obtained from the independent cardiac ultrasound unit of our cardiac center, and preoperative 24 h ambulatory ECG reports were obtained from the ECG unit of our hospital.

### Statistical Analysis

We applied a novel, modern statistical shrinkage technique, logistic least absolute shrinkage and selection operator (Lasso) regression to examine factors associated with Af recurrence. This method “penalizes” the redundant variables by introducing a penalty coefficient λ into the regression equation, i.e., gradually compressing their coefficients to zero, which can effectively identify the most relevant variables for the outcome and reduce the dimensionality of the independent variables, thus minimizing the risk of overfitting (14). In this study, 100 cycles of Lasso regression were performed for the 32 variables collected, selecting variables that were retained for all 100 times to identify factors associated with the first recurrence outcome. This method mitigates errors caused by random seeds of Lasso operations (15). The parameters were combined with clinical significance for screening. The screened variables were depicted analogously with survival curves using the Kaplan–Meier (KM) method and compared using the log-rank test for a preliminary understanding of recurrence factors.

As for establishing the prediction model, first, we combined it with the stepwise logistic regression method based on the Akaike information criterion (AIC) (16). A logistic regression model for predicting whether Af would recur within a year was created using 70% of the data as a training set and the remaining data to validate the accuracy. The efficiency of the model was evaluated by concordance index (C-index), and the discriminant performance of the model was measured using C-statistic, ranging from 0.5 (random forecast) to 1.0 (excellent distinction) (17). In the calibration plot, calibration could be envisioned. The prediction probability of the result is overestimated when the correction intercept is less than 0. In contrast, the prediction is underestimated when the intercept is positive (18, 19). Meanwhile, a decision curve analysis was performed to show the net benefit of the different models. The “None” line would display the expected net benefit if the interference changes were not performed. The “ALL” line presents the expected net benefit of developing the intervention for all patients (17).

Data analysis, curve drawing, and model establishment were performed using the R version 4.0.3 (The R Foundation, Vienna, Austria).

### Detailed Methodology

To detect the relationship between the Af and different clinical variables for our dataset, two algorithms were adopted. First is the Lasso regression for the feature selection and the second is logistic regression for the predicting model establishment.

The idea of Lasso regression is to optimize the cost function to reduce the absolute values of the coefficients. In our case, we build the model by using the R package glmnent() function and using cv.glmnent() to do the cross-validation to select the model. Besides, we set the different random seeds to run the model (100 different random seeds and perform 100 different models), we finally selected 4 variables which eventually retained 98% of models based on the optimized lambda value.

After the variable selection, the next step is to build the prediction model. In this case, we applied logistic regression to the data and used the receiver operating characteristic (ROC) curve, area under the curve (AUC) value, and confusion matrix to evaluate the model performance.

The K-fold cross-validation is used for assessing the model performance. For this, 1-fold is held out for validation while the other k-1-folds are used to train the model and then used to predict the target variable in our testing data. This process is repeated k times, with the performance of each model in predicting the hold-out set being tracked using a performance metric, such as accuracy. A 10-fold cross-validation technique is used here; it will reduce the risk of overfitting. We have 200 samples in our dataset, 70% of the data will be used to train and validate the model, and the rest of 30% data will be used for the test data. The trainControl() function and train() function from R package caret will be used here.

## Results

### Demographics, Description of the Study Population

The characteristics of the participants in this study are shown in Table 1.

**Table 1:** Characteristics of participants.

**Categorical variables**	**Values (%)**	**Continuous variables**	**Median (interquartile range)**
Male	120 (60%)	Age at first RFCA (years)	65 (58–70)
Female	80 (40%)	Age at recurrence population (years)	65 (57–69)
Hypertension	115 (57.5%)	Recurrence interval after RFCA (months)	10 (5–16)
Coronary heart disease (CHD)	38 (19%)	The duration of RFCA procedure (hours)	2.8 (1.4–3.2)
Diabetes	35 (17.5%)	The duration of Af attack time (months)	12 (2–48)
Combined with other arrhythmias	56 (28%)	RFCA energy (watt)	35 (30–35)
Combined with COPD,PH,enlarged right atrium	16 (8%)		
Cardiac dysfunction	8 (4%)	BMI	24.97(23.12–27.16)
Paroxysmal Af	151 (75.5%)	CHA_2_DS_2_-VASc	2 (1–3)
Persistent Af	49 (24.5%)	HAS-BLED	1 (1–2)
RFCA done with ICE guidance	95 (47.5%)	Left atrial diameter (mm)	36 (33–41)
Recurrence rate	19.5%	BNP (pg/mL)	153.4 (65.0–602.6)
Percentage of recurrent population with hypertension	25 (12.5%)	Troponin (ng/mL)	0.008 (0.005–0.01)
Percentage of recurrent population with CHD	11 (5.5%)	High-density lipoprotein (mmol/L)	1.13 (0.97–1.28)
Percentage of recurrent population with Diabetes	8 (4%)	Low density lipoprotein-C (mmol/L)	2.15 (1.52–2.72)
Percentage of recurrent population combing with other arrhythmias	9 (4.5%)	Homocysteine(μmol/L)	11.8 (9.88–14.93)
		Albumin(g/L)	15 (11–23)
		Prothrombin time(s)	96 (75–124)
		INR	3.9 (2.7–5.9)
		Activated partial thrombin time(s)	22 (18–30)
		Thrombin time(s)	191 (172–233)
		Fibrinogen(g/L)	4.6 (4.05–5.25)
		D-Dimer	0.21(0.15–0.39)

*COPD: chronic obstructive pulmonary disease; PH: pulmonary hypertension; BMI: body mass index*.

### Lasso Regression for Recurrence Factors

**Figure 3:**
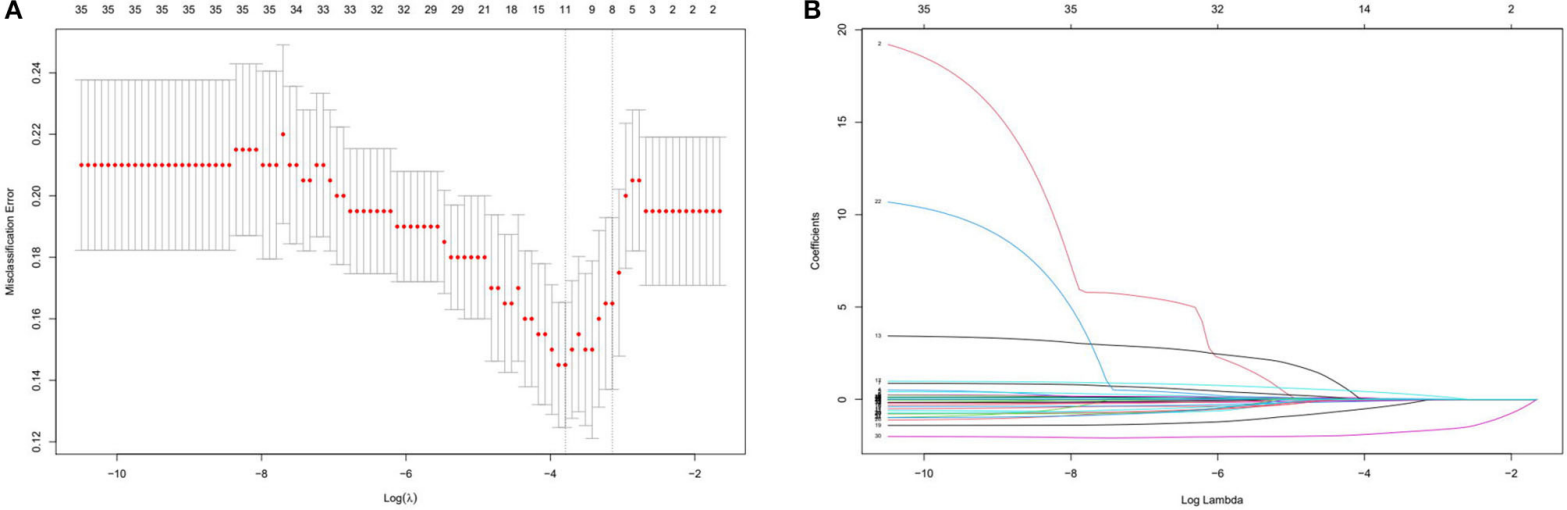
This figure shows the results of a one-time randomized screening by least absolute shrinkage and selection operator (Lasso) regression. **(A)** Variation of misclassification error. The horizontal axis shows logλ and the vertical axis shows the misclassification error. The numbers above the curve represent the number of feature of nonzero coefficient. The left dotted line represents the feature number corresponding to 0 standart error of misclassification. **(B)** The shrinkage plot of coefficients. The horizontal axis shows logλ and the vertical axis shows coefficient. The numbers above the curve represent number of features with nonzero coefficient.

The left atrial diameter (millimeter), albumin (gram per Liter), type of Af, duration of RFCA procedure (hours), duration of Af attack time (months), whether combined with other arrhythmias and ablation energy were all selected by the Lasso regression; thus, we only listed the weights accounting for one time, as shown in Table 2. Figure 3 shows the results of a one-time randomized screening by Lasso regression.

**Table 2:** The least absolute shrinkage and selection operator (Lasso) regression screening for atrial fibrillation (Af) recurrence outcome performed once at random.

**Categories**	**Coefficient**
Left atrial diameter(mm)	0.036245881
Albumin(g/L)	−0.048939977
The type of Af	1.774478486
The duration of RFCA procedure (hours)	0.317161917
The duration of Af attack time(months)	0.001257498
Whether combined with other arrhythmias	−0.276410645
Ablation energy	−0.057912708

*This is the result of a random screening performed by Lasso regression; we only listed the performance of the parameters performed 100 times and selected 100 times in this screening; the remaining variables were omitted. As shown in the table, the weight of the Type of Af remained significant*.

**Figure 4:**
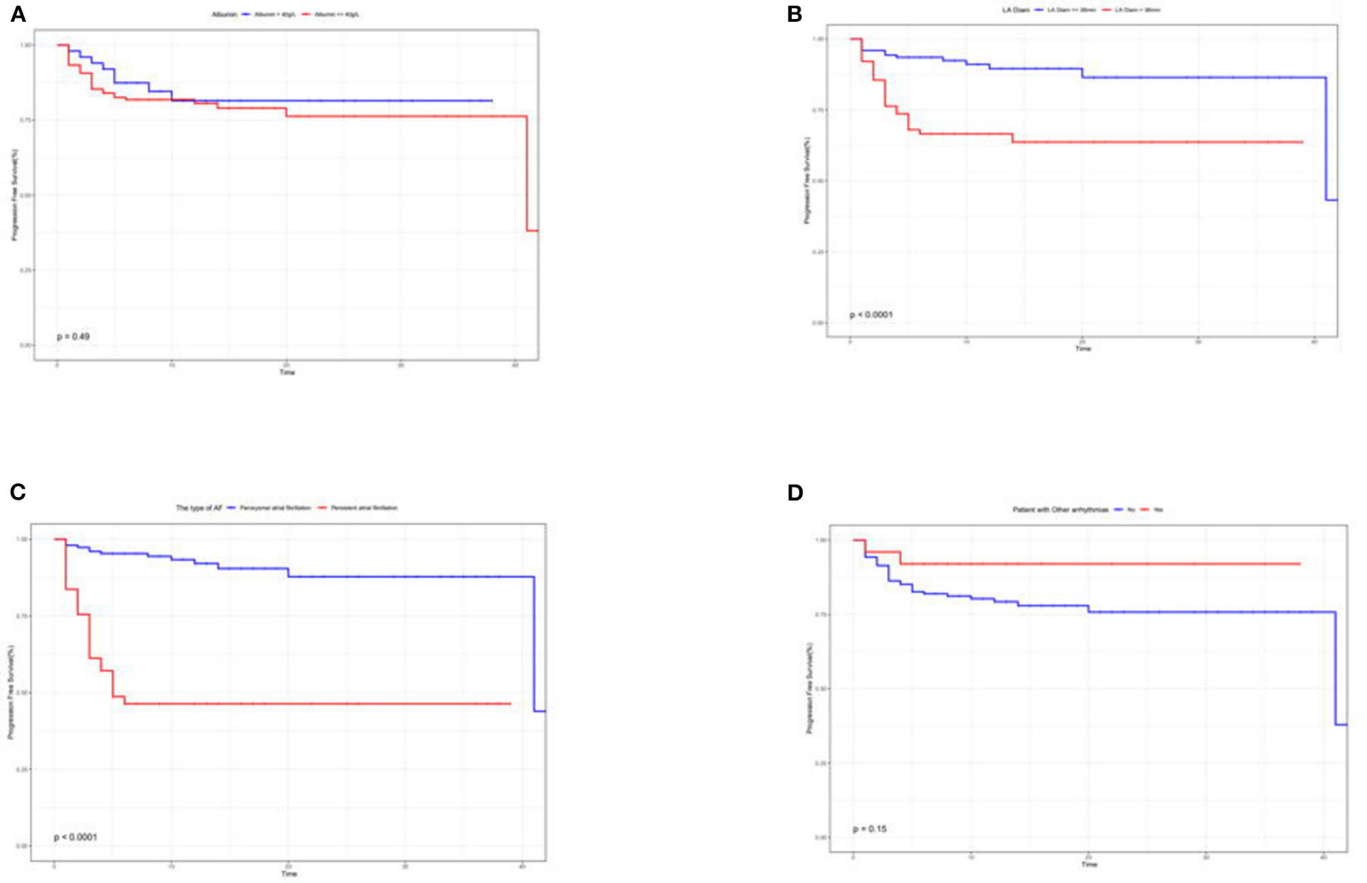
**(A–D)** Kaplan–Meier survival curves to assess the impact of albumin **(A)**, left atrial diameter **(B)**, type of Af **(C)**, and whether other arrhythmias combined **(D)** on the recurrence prognosis of patients.

Kaplan–Meier curves illustrated that the type of Af and left atrial diameter significantly affected the recurrence rate (Figures 4A–D).

### A 1-Year Logistic Regression Prediction Model

The following equation was derived from logistic regression:


$$\begin{array}{l} p=\frac{1}{1+\text{exp}\left(-R\right)} \end{array}$$

Where *R* = 4.046703 + 0.065109 LA – 0.174490 albumin – 2.290462 the type of Af + 0.004854 time – 0.536535 hypertension + 0.485112 diabetes + 0.482281CHD (Table 3).

**Table 3:** Modeling results using logistic regression.

** 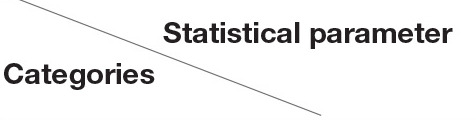 **	**Estimate**	**Standard error**	**Z value**	**Pr(>| Z |)**

LA (mm)	4.404673	3.956402	1.023	0.3064
Albumin (g/L)	0.06591	0.042474	1.533	0.1253
Time (months)	0.004854	0.002461	1.972	0.0486
The type of Af	−2.290462	0.549872	−4.165	0.0000311
Hypertension	−0.536535	0.524952	−1.022	0.3068
Diabetes	0.485112	0.624757	0.776	0.4375
Coronary heart disease	0.482281	0.580627	0.831	0.4062

*LA: left atrial diameter (mm); albumin (g/L), the type of Af; paroxysmal Af = 1, persistent Af = 0; time: the duration of Af attack time (months); hypertension None = 0,Yes = 1; diabetes None = 0, Yes = 1; CHD: coronary heart disease None = 0, Yes = 1. Among these, the p of the duration of Af episode and the type of Af were less than 0.05*.

In this formula, *P* = probability of recurrence. According to statistical principles, a cut-off greater than 0.5 is considered to be a recurrence.

**Figure 5:**
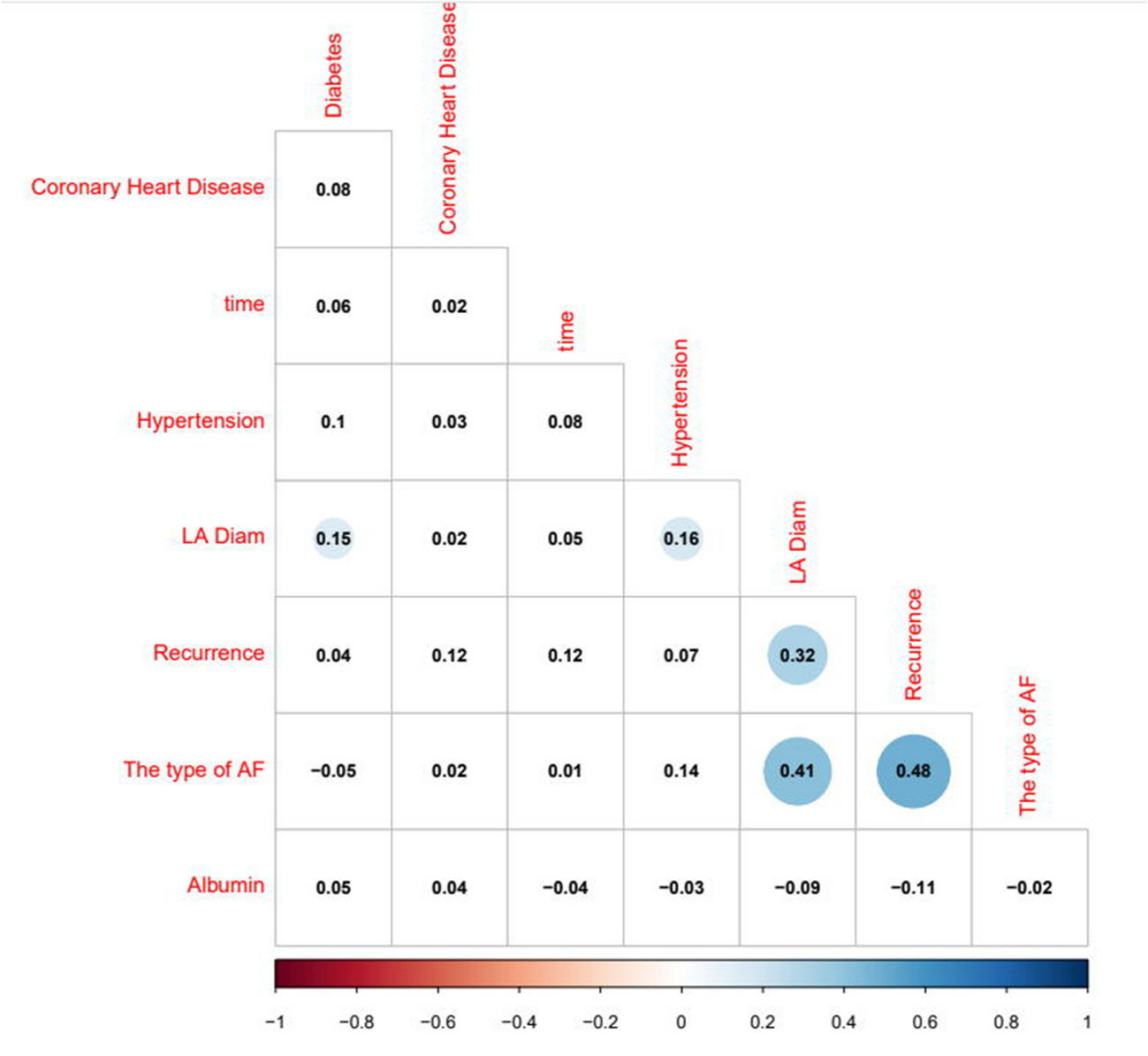
Heatmap of correlation coefficients after logistic regression modeling.

**Figure 6:**
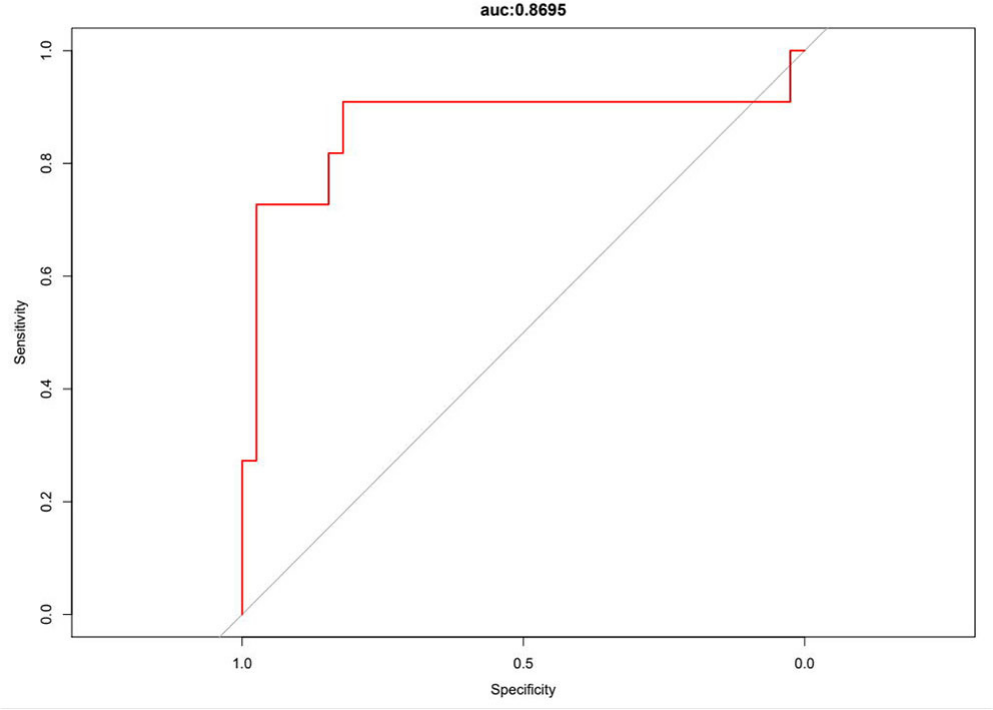
Receiver operating characteristic (ROC) curve of the prediction model.

**Figure 7:**
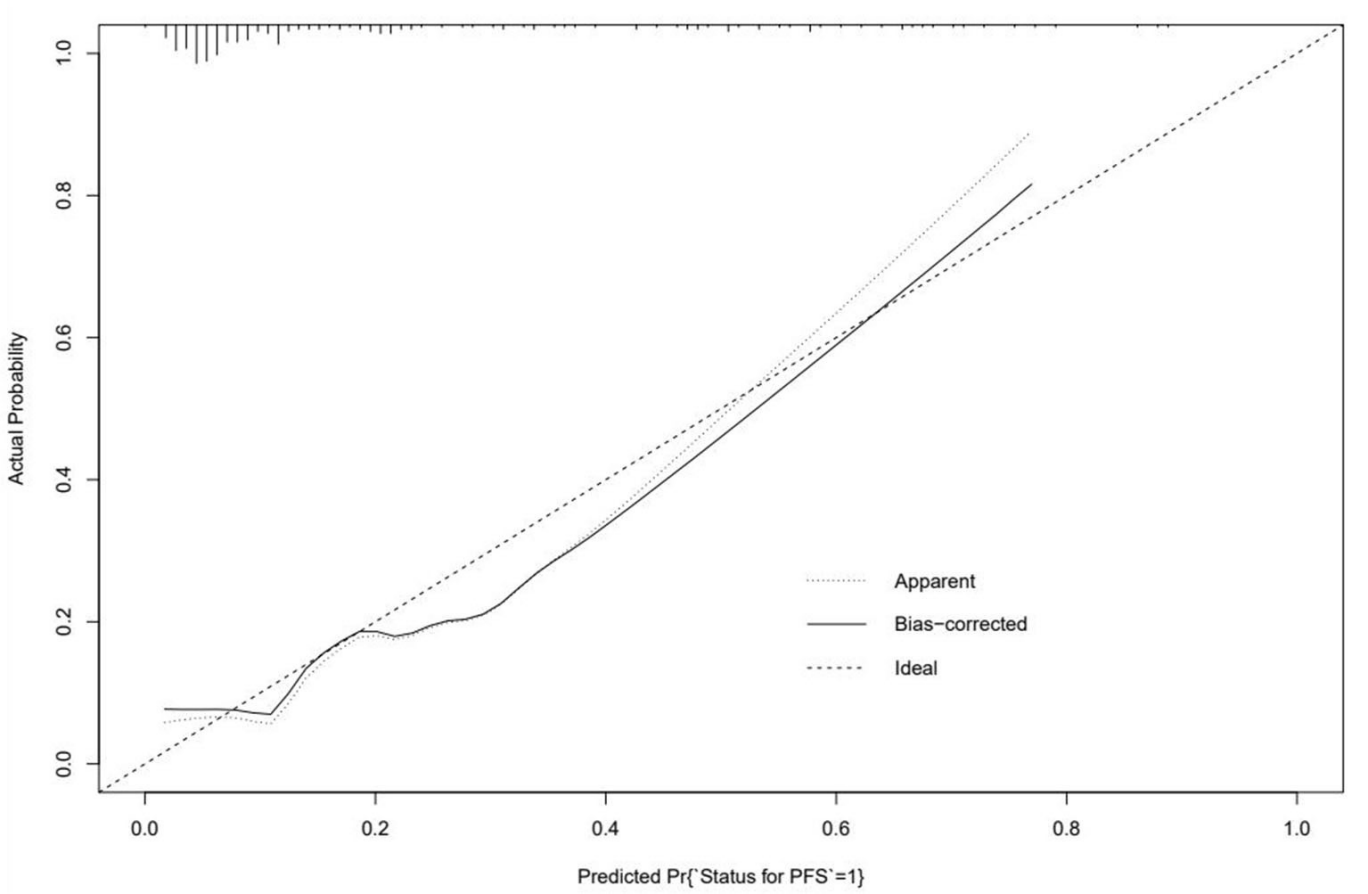
Calibration curve of the prediction model.

**Figure 8:**
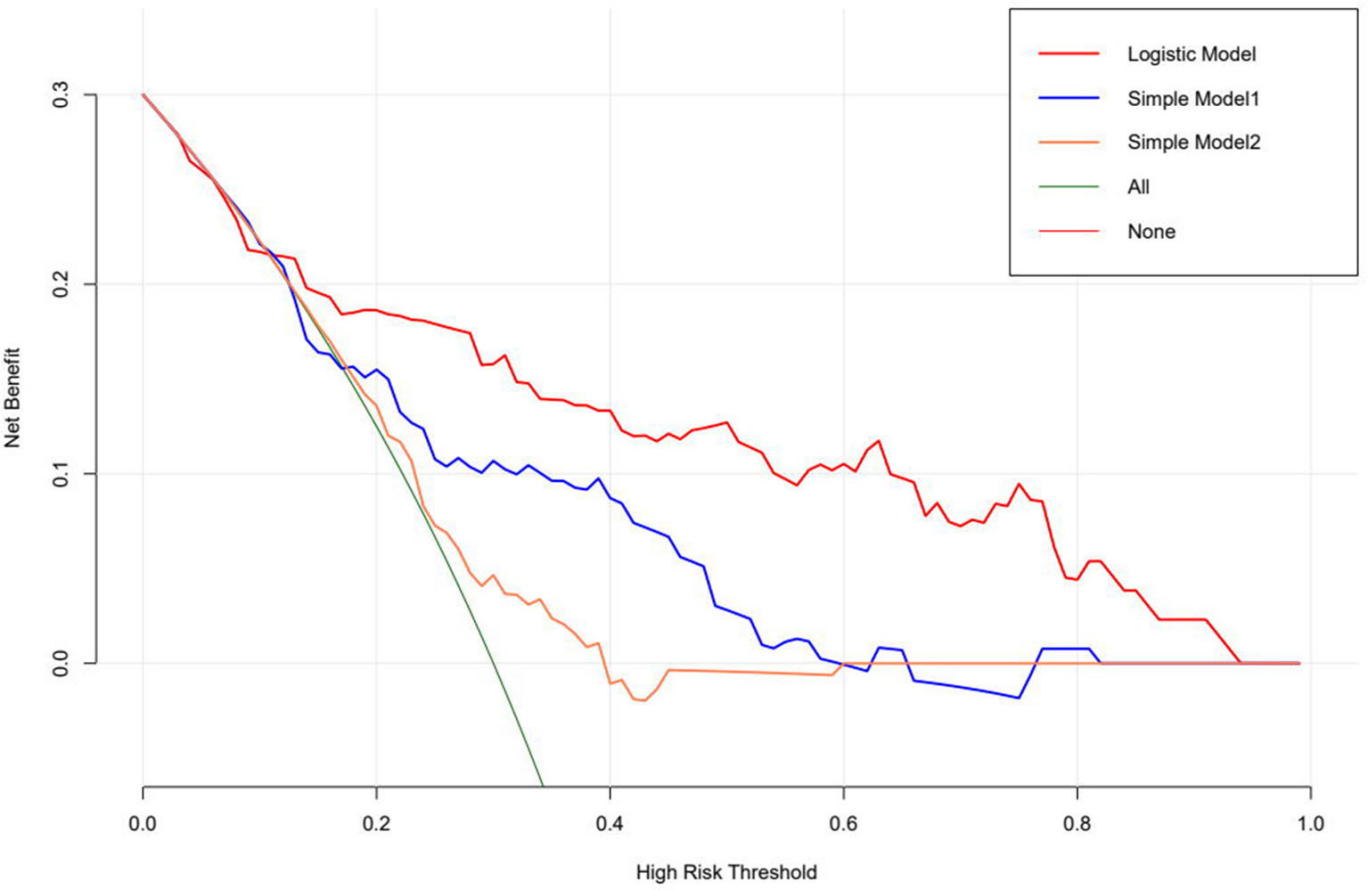
Clinical decision curve.

The logistic regression modeling prediction results are shown in Table 4. Then we drew a heat map of correlation coefficients after logistic regression modeling as shown in Figure 5. And we plotted the ROC curve, calibration curve and clinical decision curve of the prediction model as shown in Figures 6–8, respectively.

**Table 4:** Logistic regression modeling prediction results.

** 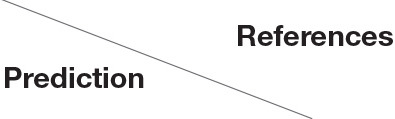 **	**0**	**1**

0	38	3
1	1	8

*Accuracy: 0.92(95% CI 0.8077, 0.9788); Kappa: 0.7506; sensitivity: 0.7273; specificity: 0.9744*.

## Discussion

### Background and Rationale

The mechanism of Af has been researched by many scholars but yet to be elucidated. At present, the most popular academic view is the pulmonary vein potential theory, so the aim of ablation is to isolate the pulmonary vein potential. However, the treatment effect is not ideal, and the 1-year recurrence rate can reach 30–50% (3, 4). Furthermore, to minimize the postoperative recurrence rate and improve the quality of life of patients with Af, this study gathers unique insights by using preoperative biological index information. This provides a deeper understanding of the treatment strategy of this challenging disease and could improve the ablation treatment outcomes. Therefore, a retrospective analysis of patients who completed Af RFCA for the first time in our hospital was carried out.

### Risk Factors for Recurrence Factors

Statistically, as long as patients have completed Af ablation at our center in 2020, without this study exclusion criteria, the 1-year recurrence rate after the ablation procedure was 28%. Rigorous measures were taken to ensure that Af RFCA was performed with high quality. The recurrence interval after RFCA was 10 (5–16) months, and the recurrence rate reached 19.5%. General information about the ablation can be found in Table 1.

Under this favorable context, we have made the following reflections on the high recurrence rate. The screened indicators were divided into two dimensions according to clinical features and laboratory factors.

First, the type of Af determines the ablation prognosis (20). Either the results screened by Lasso regression or the correlation heatmap analysis coincides with the perception obtained from the CA experience of our team. Margulescu AD et al. have reported that different types of Af should be treated and managed differently in clinical practice (6). Moreover, statistical differences were demonstrated in the K–M survival curves in patients with recurrent outcomes. On account of the insidious nature of atrial fibrillation, timely diagnosis in its initial stages is very challenging (21, 22). Most patients do not seek medical attention until they experience symptoms, such as panic, palpitation, dizziness, weakness, and chest tightness. It is only at the hospital that an ECG or 24-h ambulatory ECG is performed to capture an episode of Af. In cases of paroxysmal Af, the diagnosis cannot be made solely based on ancillary tests. When the patient is not experiencing an attack, the ECG is almost identical to that of a normal person. However, missing the early diagnosis and treatment significantly affects the treatment prognosis. For the above reasons, we have also included the duration of Af attack time, but this variable is based solely on the chief complaints of the patient. Consequently, the duration of symptoms was faithfully recorded in the electronic medical record system without an objective assessment. As expected, the duration of Af episodes in our analysis was also identified as a prognostic indicator. The potential causes indicate that the duration of the Af attack time is related to myocardial remodeling (23). Furthermore, the left atrial diameter changed in response to irregular fibrillation waves. If interventions are not performed as soon as a clear diagnosis is available, the type of Af can change, and the prognosis will be affected (6, 20, 24).

As for patients with the presence of concurrent arrhythmias,we have found that the prognostic outcome is better than not being suffered from co-arrhythmias. Johnson et al. used supraventricular tachycardia as an indicator to predict the onset of Af. Based on the ablation experience of our team, inhibiting the feedback loop in a pre-excitation syndrome of Af patients naturally caused the Af to disappear. However, this mechanism was not explored in this study and remained an observation at the macroscopic level (25). Shin et al. observed a significant association between end-stage progression of chronic obstructive pulmonary disease (COPD) and the prognostic outcome of Af in patients suffering from both diseases. In addition, Liu YX and Wu C et al. found an inextricable association between pulmonary hypertension and Af. The absence of a significant association in our current study may be related to the patients with COPD not being in the terminal stage, and some patients with severe disease were excluded from the study (26–28).

Hypertension, coronary artery disease, diabetes, and body mass index (BMI) have been reported to be independent factors associated with Af recurrence (20, 28–36). They may have been partially screened in the Lasso regression, and they all could not be identified in this sample. As for the above results,we mainly consider that they are caused by the small sample size or inclusion bias, but we would include the consensus indicators in the follow-up prediction model to make it more robust and generalizable. However, we did not include BMI in the follow-up modeling because BMI reflects the nutritional status of the patients. Unlike albumin, BMI was not screened by the Lasso regression. Considering that repeated significance variables usually lead to the overfitting of the model, we finally removed it.

In serology, only albumin was associated with the prognostic outcome. Albumin has been shown by the team of Dianne E to be an independent predictor, and it is even superior in critical conditions (37). Albumin reflects a certain extent on the nutritional status of the body. Expectedly, a good nutritional status leads to a superior response to surgical stress (38). However, in the K–M survival curves, no statistically significant difference in albumin levels were found in patients with or without recurrence. We prefer an enrollment bias, enrolling hospitalized patients of relatively advanced age, and the older they are, the lower are their albumin levels (39). Some patients have a poorer medical foundation than patients of the same age group.

In this study, we included CHA2DS2-VASc, HAS-BLED, BNP, troponin, coagulation indicators, high-density lipoprotein (HDL), low-density lipoprotein cholesterol (LDL-c), homocysteine, and many other unanimously accepted indicators that are directly or indirectly relevant in terms of cardiovascular disease (20, 40, 41). To ensure that these indicators are appropriately screened, a rigorous confirmation of indicators linked to liver and kidney function was carried out to minimize the influence on the variable outcomes. Therefore, we did not gather the creatinine and liver enzyme levels. BNP and troponin are usually influenced by renal function, and the remaining indicators are closely related to liver function (42–44). Unfortunately, the above-mentioned indicators were not selected in the Lasso regression. We have concluded the following:

I) The laboratory department of our hospital usually marks “less than a certain threshold value ensuring normal” for patients with normal BNP or troponin indicators. We used this threshold value in the analysis, which is not a specific value.
II) Exclusion criteria and admission bias: we have excluded patients with acute chest pain or New York Heart Association (NYHA) III or higher from this study, reflecting that they did not have preoperative manifestations of myocardial-related damage in lateral. Indicators, such as CHA2DS2-VASc and HAS-BLED, are highly correlated with coagulation, but both were not selected. To some extent, this reflects that the results of our analysis are reasonable (12, 13, 45).
III) Differences in the indicators themselves: the therapy for statins has been reported to reduce the probability of Af recurrence. HDL is highly related to the prognosis of CHD, and a similar relationship exists between homocysteinemia and some patients with hypertension (41, 46). However, their effect on recurrence in Af may be indirect. Shang Y et al. confirmed that lipid levels and recurrence outcomes were not independently associated (40). The dichotomous variables, such as the presence or absence of hypertension, coronary artery disease, and diabetes were not included in this Lasso analysis. Understandably, these indirectly related indicators were not identified.

Besides, we briefly included a statistical analysis of the procedure [whether it was done under intracardiac echocardiography (ICE) guidance] and the duration of the procedure that was not surprisingly included in the recurrence outcome. After 2018, Af ablation at our institution was essentially done under ICE guidance. However, these two items are strongly influenced by subjective operator factors and objective external conditions, and we will not expand on them here.

### A 1-Year Logistic Regression Prediction Model

We constructed a prediction model of whether Af would recur within 1 year for all patients with a recurrent outcome (*n* = 39). We elected independent variables identified in Lasso regression with higher clinical significance and combined some independent variables previously approved by scholars to maximize the predictive power for generalizability. Our predictions can be used as a reference for clinical physicians to improve the prognosis of patients before ablation. The logistic regression prediction model has an excellent performance in imaging (47).

In this model, the correlation coefficients in front of each variable provided a rough perception of the weight of each variable, and a correlation heatmap was plotted accordingly. The type of Af is the most obvious. In addition, if we use the logistic regression model to predict “1,” we take aggressive treatment of the primary disease (e.g., hypertension and coronary artery disease) and improve preoperative nutritional status to minimize the recurrence rate before ablation in patients with recurrent outcomes. For patients with a prediction of “0,” we recommend ablation. The optimistic corrected C statistic for this model was 0.83 (95% *CI* 0.56–1), and the ROC was 0.8695 (95% *CI* 0.821, 0.909). Calibration plots showed good overall agreement between predicted and observed 1-year outcomes, presenting a clear correlation between the predicted and actual observation.

Besides, we used decision curve analysis to demonstrate our model. The results of our model are very satisfactory.

### Limitation

This study has some unavoidable limitations. First, it was a single-center retrospective study with a limited sample size. We were unable to perform external validation to demonstrate the generalizability of our model since no one has performed such a novel prediction study in Af recurrence outcome. Second, our definition of Af recurrence and the timing of Af episodes were also flawed. This study only showed a potential association between preoperative-related variables and prognosis. Some of the variables identified in previous studies were not well quantified, such as blood pressure values and left ventricular ejection fraction but were simply dichotomized as “yes or no” variables. Moreover, we have taken a macroscopic view and cannot explain the specific mechanisms because our study aimed to find indicators that could predict the outcome. Although we have included a relatively large number of variables, there may be some potential variables that could incur ignored recurrence outcomes.

Due to sample size issues, we included all patients with Af recurrent outcomes in the model construction, regardless of whether the recurrence interval fell within 1 year. The inclusion bias of the sample size resulted in some previously recognized indicators not being included. We only focused on the recurrence outcome within 1 year and did not take into account any further development. Moreover, it is only a mathematical model, and although the coefficient has a certain clinical significance and reference value, it cannot be comprehensively analyzed in the formula. In addition, although the ablation energy and the duration of RFCA were independent determinants of patient outcomes in this sample, multiple procedures, such as cryoablation were not included in the analysis due to the relative expertise of our center in RFCA. Furthermore, operator experience and improvement of equipment were not discussed.

## Conclusion

In conclusion, we found that the left atrial diameter, albumin, the type of Af, whether other arrhythmias were combined, the duration of Af attack time were significantly associated with Af recurrence. Several clinical features and serologic markers can predict the outcome of recurrence within 1 year after Af RFCA. The model can guide preoperative evaluation and clinical decision-making. More importantly, it can potentially improve recurrence outcomes.

## Data Availability Statement

The raw data supporting the conclusions of this article will be made available by the authors, without undue reservation.

## Ethics Statement

The studies involving human participants were reviewed and approved by Ethics Committee of the Fourth Affiliated Hospital of Zhejiang University School of Medicine. Written informed consent for participation was not required for this study in accordance with the national legislation and the institutional requirements.

## Author Contributions

SJ and HM: substantial contributions to the conception or design of the work. SJ, YW, WL, YZ, YiC, YaC, QZ, WW, and CF: the acquisition, analysis or interpretation of data for the work. SJ: drafting the work or revising it critically for important intellectual content. SX: provide approval for publication of the content and agree to be accountable for all aspects of the work in ensuring that questions related to the accuracy or integrity of any part of the work are appropriately investigated and resolved. All authors contributed to the article and approved the submitted version.

## Funding

The paper was supported by the National Natural Science Foundation of China (No. 81971688).

## Conflict of Interest

The authors declare that the research was conducted in the absence of any commercial or financial relationships that could be construed as a potential conflict of interest.

## Publisher's Note

All claims expressed in this article are solely those of the authors and do not necessarily represent those of their affiliated organizations, or those of the publisher, the editors and the reviewers. Any product that may be evaluated in this article, or claim that may be made by its manufacturer, is not guaranteed or endorsed by the publisher.
